# Novel standardized indexes of brainstem auditory evoked potentials for predicting hearing preservation in vestibular schwannomas

**DOI:** 10.1038/s41598-024-58531-8

**Published:** 2024-05-08

**Authors:** Liwu Jiao, Xuyang Liu, Hongtao Zhu, Chao Guo, Junwen Wang, Kai Shu

**Affiliations:** Department of Neurosurgery, Tongji Hospital, Tongji Medical College, Huazhong University of Science and Technology, Wuhan, 430030 China

**Keywords:** Neuroscience, Auditory system, Diseases of the nervous system

## Abstract

Hearing preservation (HP) during vestibular schwannomas (VSs) surgery poses a significant challenge. Although brainstem auditory evoked potentials (BAEPs) on the affected side are commonly employed to monitor cochlear nerve function, their low signal-to-noise ratio (SNR) renders them susceptible to interferences, compromising their reliability. We retrospectively analyzed the data of patients who underwent tumor resection, while binaural brainstem auditory evoked potentials (BAEPs) were simultaneously recorded during surgery. To standardize BAEPs on the affected side, we incorporated the synchronous healthy side as a reference (interval between affected and healthy side ≤ 3 min). A total of 127 patients were enrolled. Comparison of the raw BAEPs data pre- and post-tumor resection revealed that neither V-wave amplitude (Am-V) nor latency (La-V) could serve as reliable predictors of HP simultaneously. However, following standardization, V-wave latency (STIAS-La-V) and amplitude (STIAS-Am-V) emerged as stable predictors of HP. Furthermore, the intraoperative difference in V-wave amplitude (D-Am-V) predicted postoperative HP in patients with preoperative HP and remained predictive after standardization. The utilization of intraoperative synchronous healthy side BAEPs as a reference to eliminate interferences proves to be an effective approach in enhancing the reliability of BAEPs for predicting HP in VSs patients.

## Introduction

Vestibular schwannomas (VSs), accounting for 8% of all intracranial tumors and the most common neoplasm of the Cerebellopontine Angle (CPA), are benign tumors deriving from myelinating Schwann cells of the vestibular branch of the vestibulocochlear (eighth cranial) nerve^1,2^. Microsurgical resection currently stands as the sole radical and frequently preferred treatment option with low mortality rates (< 0.5%) and less than 10% risk of permanent facial weakness^3,4^. However, hearing preservation (HP) has always posed a significant challenge for physicians and patients alike; postoperative HP in VSs is less than one-third compared to over 70% before surgery^5–7^. This decline can be primarily attributed to intraoperative cochlear nerve injury, traction and nerve sheath manipulation or vascular ischemia, reliable intraoperative neuromonitoring techniques are essential in avoiding these intraoperative injuries^8^.

Methods traditionally used for intraoperative cochlear nerve monitoring include brainstem auditory evoked potential (BAEPs), cochlear nerve action potential (CNAP), and transotympanic electrocochleography (TT-ECochG)^9^. CNAP can realize continuous real-time monitoring of cochlear nerve function during surgery, but it requires clear anatomical positioning of cochlear nerve and space for placing electrodes, so it is mostly used for VSs with small tumor size (< 1.5 cm)^10,11^. TT-ECochG is an invasive procedure that requires the insertion of recording electrodes through the tympanic membrane to reflect the cochlear nucleus and the external cranial segment of the auditory nerve, so it is mainly used for surgery involving the inner ear and/or cochlea^12^. At present, BAEPs is the most widely used technology for monitoring cochlear nerve function during surgery in the CPA^13,14^. In addition to its simple operation, the prognostic significance of intraoperative BAEPs for HP, particularly during VSs resection, has been widely acknowledged, and distinct indicators of cochlear nerve injury such as the disappearance of stable waves, delay of V wave latency, and decrease of V wave amplitude have been identified^15–17^. However, the limited reliability of raw indicators from intraoperative BAEPs arises due to poor signal-to-noise ratio (SNR) and various intraoperative interferences leading to significant variations in sensitivity and specificity for predicting HP across different studies ranging from 37 to 100%^18^. Previous studies have predominantly focused on analyzing BAEPs solely from the affected side while neglecting the healthy side^9,19^. Nevertheless, theoretically incorporating heathy side BAEPs from the synchronously recorded binaural BAEPs as a reference can effectively eliminate interferences. Therefore, this study aims to retrospectively analyze intraoperative BAEPs and standardize those obtained from the affected side by utilizing the healthy side as a reference in order to identify reliable indicators that can predict HP accurately and guide surgeons towards making decisions more conducive to HP during VS resection.

## Results

### Patient characteristics

A total of 127 patients were enrolled in the present study (Table 1). The mean age of patients at surgery was 48 years (range 15–68 years), with 76 patients (59.8%) being female. Furthermore, 65 patients (51.2%) were found to have tumors on the left side. The mean duration of symptoms (DOS) for VSs was 27.3 months (range 0.5–240 months). The average tumor diameter was measured to be 30.9 mm (range 12–76 mm). Among the patient cohort, a total of 79 (62.2%) were classified as Koos grade IV, while smaller proportions exhibited grade III and grade II, consisting of 12 (9.4%) and 26 (20.5%) patients respectively. Goss total tumor resection was performed in all but one case (99.2%).

**Table 1:** Demographics, tumor characteristics, and audiometric outcomes of the study population.

Characteristic	Mean ± SD (range) or No. (%)
Age (years)	48 ± 13 (15–68)
Female	76 (59.8)
Left side	65 (51.2)
DOS (mos)	27.3 ± 43.4 (0.5–240)
Tumor diameter (mm)	30.9 ± 9.7 (12–76)
≤ 20	16 (12.6)
20–30	41 (32.3)
30–40	50 (39.4)
> 40	20 (15.7)
Koos grade	
Grade II	12 (9.4)
Grade III	26 (20.5)
Grade IV	79 (62.2)
Preoperative AAO-NHS classification	
Class A	29 (22.8)
Class B	36 (28.3)
Class C	19 (15.0)
Class D	43 (33.9)
Preoperative hearing preservation	
Preserved	84 (66.1)
PTA (dB)	33.9 ± 17.0 (10–67)
WRS (%)	66.0 ± 10.9 (51–90)
Not preserved	43 (33.9)
Postoperative AAO-NHS classification	
Class A	8 (9.5)
Class B	12 (14.3)
Class C	18 (21.4)
Class D	46 (54.8)
Postoperative hearing preservation	
Preserved	38 (45.2)
PTA (dB)	43.3 ± 18.5 (11–70)
WRS (%)	63.6 ± 6.7 (50–83)
Not preserved	46 (54.8)

### Hearing outcomes

**Figure 1:**
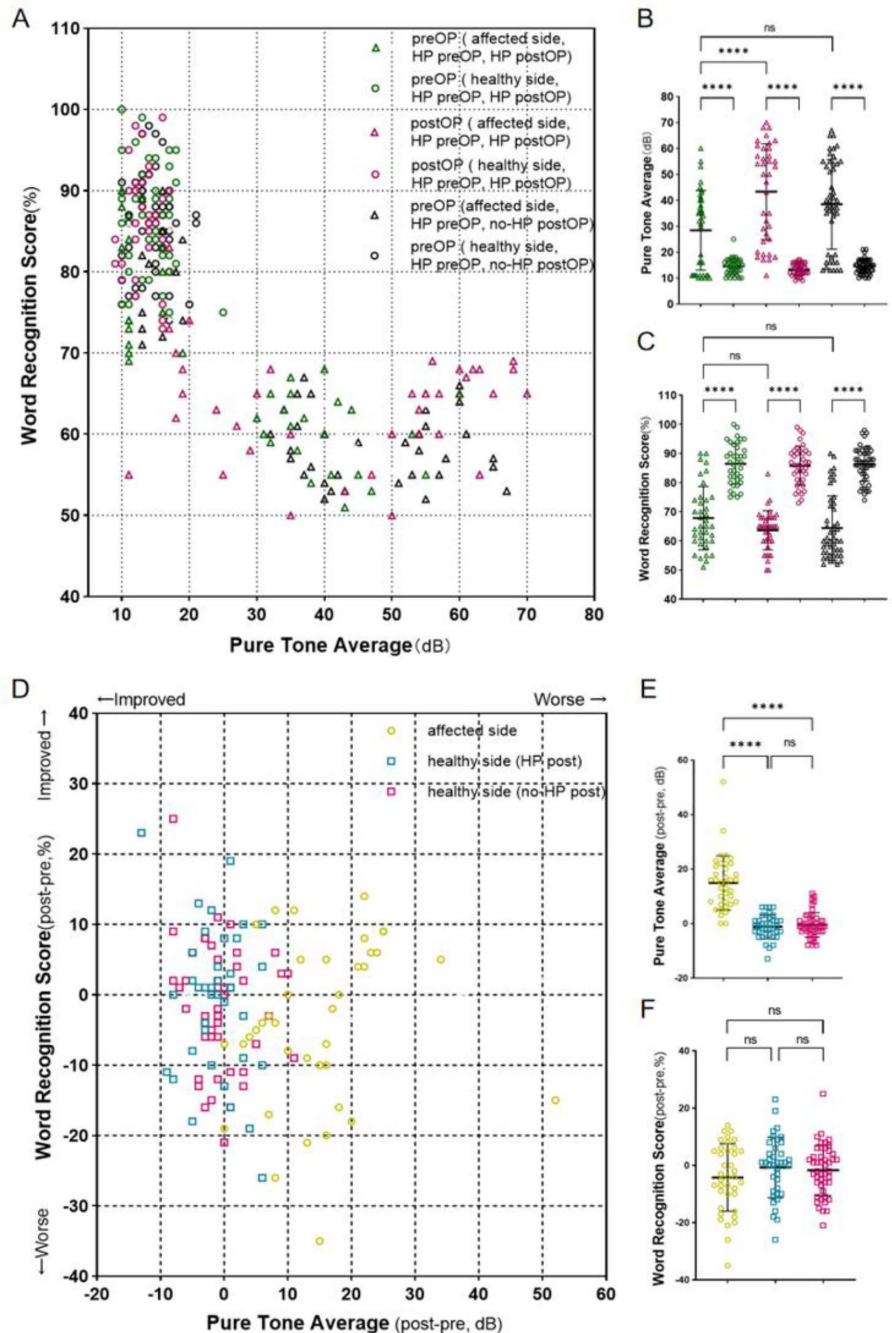
(**A**) Scattergram demonstrating PTA and WRS in all patients with HP (n = 84), PTA (**B**) and WRS (**C**) between different groups, (**D**) Scattergram demonstrating PTA and WRS changes (post operation-pre operation) in patients with post-operation HP (n = 38), PTA (**E**) and WRS (**F**) changes (post operation–pre operation) between different groups. *Significant difference (P < 0.05), ns No significant difference (P > 0.05).

Preoperative evaluation showed that 84 patients (66.1%, 84/127) had HP, the mean PTA was 33.9 ± 17.0 dB (range 10–67 dB), and the mean WRS was 66.0 ± 10.9% (range 51–90%). 29 cases (22.8%) were classified as AAO-NHS class A, 36 cases (28.3%) as class B, and 19 cases (15.0%) as class C. Following resection, 38 patients (29.9%, 38/127) had HP, the mean PTA was 43.3 ± 18.5 dB (range 11–70 dB), and the mean WRS was 63.6 ± 6.7% (range 50–83%). 8 cases (9.5%) classified as AAO-NHS class A, 12 cases (14.3%) as class B, and 18 cases (21.4%) as class C. Hearing scattergrams are shown in Fig. 1.

The analysis of WRS and PTA test results in each group found that there were statistical differences between the affected and healthy side. In the patient cohort (HP pre-operation), grouping of HP outcomes after surgery (HP post-operation vs. no-HP post-operation) showed no difference in PTA (P = 0.07) and WRS (P = 0.41). In addition, we analyzed the changes of PTA and WRS before and after operation and found that there was no statistical difference (P = 0.89 and P = 0.91 respectively) before and after operation on the healthy side. Compared with the healthy side, the changes of PTA in the affected side was significantly increased (P < 0.01), but the changes of WRS was not significant (P > 0.05).

### Pre-resection BAEPs

**Figure 2:**
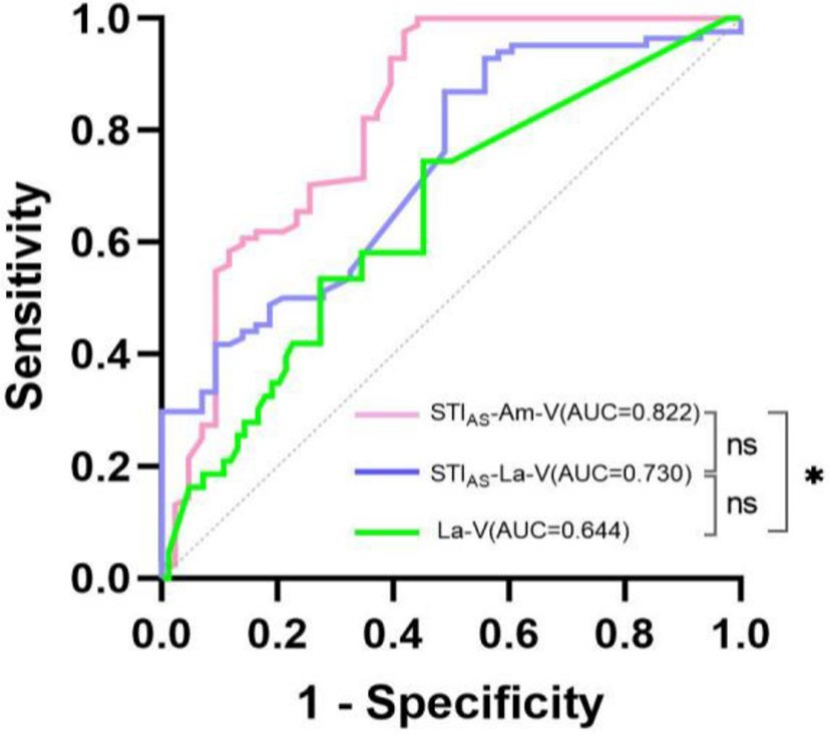
A receiver operating characteristic (ROC) curve of pre-resection BAEPs predictors of HP, *Significant difference (P < 0.05), ns No significant difference (P > 0.05).

Among the 84 patients (66.1%, 84/127) with preoperative HP, logistic regression analysis revealed that La-V (OR 0.343, P = 0.031) was a negative predictor of preoperative HP among the raw pre-resection BAEPs indicators. After normalization, univariate analysis identified four predictors of HP: STI_AS_-La-I (OR 0.014, P = 0.001), STI_AS_-La-V (OR < 0.001, P < 0.001), STI_AS_-La-III-V (OR 0.346, P = 0.018) , and STI_AS_-Am-V (OR 1.858, P = 0.013). Further multivariate analysis confirmed that both STI_AS_-La-V (OR < 0.001, P < 0.001) and STI_AS_-Am-V (OR 2.570, P = 0.006) were independent predictors of preoperative HP (Table 2). ROC curve analysis demonstrated an AUC of 0.822 for STI_AS_-Am-V and − 0.89 μV constituted the cutoff value with 97.6% sensitivity and 58.1% specificity. The AUC for STI_AS_-La-V was 0.737 with a cutoff value of 0.05 μV yielding a sensitivity of 86.9% and a specificity of 51.2%. While the AUC for La-V was 0.645, and ROC curve analysis revealed that the predictive accuracy of STI_AS_-Am-V was significantly superior than that of La-V (P = 0.02) (Fig. 2).

**Table 2:** Univariate and multivariate analyses of BAEPs for predicting preoperative hearing preservation.

Variables	Univariate analysis			Multivariate analysis		
	OR	(95% CI)	*P* value	OR	(95% CI)	*P* value
Raw BAEPs						
La-I (ms)	0.378	(0.075–1.904)	0.239	–	–	–
La-II (ms)	0.257	(0.055–1.198)	0.084	–	–	–
La-III (ms)	0.660	(0.189–2.302)	0.514	–	–	–
La-IV (ms)	1.133	(0.411–3.125)	0.809	–	–	–
La-V (ms)	0.343	(0.130–0.906)	**0.031**	–	–	–
La-I-III (ms)	1.208	(0.347–4.203)	0.767	–	–	–
La-III-V (ms)	0.522	(0.243–1.120)	0.095	–	–	–
La-I-V (ms)	1.250	(0.890–1.755)	0.198	–	–	–
Am-V (μV)	29.776	(0.508–1744.929)	0.102	–	–	–
Am-I (μV)	0.281	(0.024–3.213)	0.307	–	–	–
Standardized BAEPs						
STI_AS_-La-I	0.014	(0.001–0.160)	**0.001**	0.224	(0.011–4.634)	0.333
STI_AS_-La-II	0.048	(0.002–1.348)	0.074	–	–	–
STI_AS_-La-III	0.300	(0.007–12.065)	0.523	–	–	–
STI_AS_-La-IV	11.635	(0.139–970.708)	0.277	–	–	–
STI_AS_-La-V	< 0.001	(0.000–0.001)	**< 0.001**	< 0.001	(0.000–0.001)	**< 0.001**
STI_AS_-La-I-III	6.215	(0.592–65.196)	0.128	–	–	–
STI_AS_-La-III-V	0.346	(0.143–0.833)	**0.018**	0.473	(0.146–1.534)	0.212
STI_AS_-La-I-V	1.391	(0.333–5.806)	0.651	–	–	–
STI_AS_-Am-V	1.858	(1.138–3.035)	**0.013**	2.570	(1.314–5.030)	**0.006**
STI_AS_-Am-I	1.271	(0.907–1.781)	0.163	–	–	–

Significant values are in bold.

### Post-resection BAEPs

**Figure 3:**
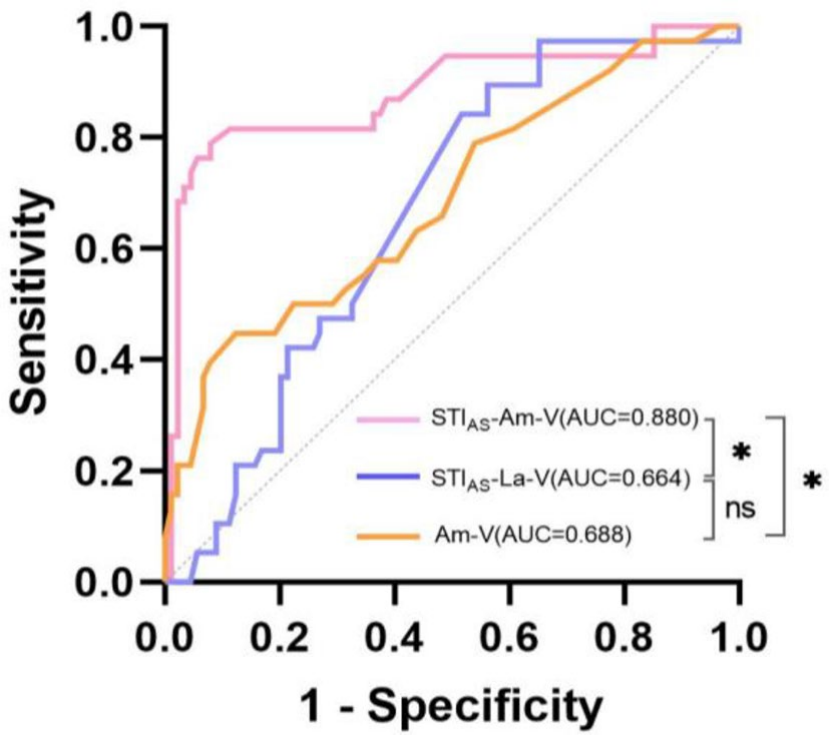
A receiver operating characteristic (ROC) curve of post-resection BAEPs predictors of HP, *Significant difference (P < 0.05), ns No significant difference (P > 0.05).

Postoperative HP was observed in 38 patients (29.9%, 38/127). Logistic regression analysis revealed that Am-V (OR 1703.860, P < 0.001) served as a positive predictor of postoperative HP among the raw post-resection BAEPs indicators. Following normalization, univariate analysis demonstrated that STI_AS_-La-V (OR < 0.001, P < 0.001) and STI_AS_-Am-V (OR 1.858, P = 0.013) were predictors of postoperative HP, and multivariate analysis also showed that both had predictive effect (Table 3). ROC curve analysis showed that the predictive accuracy of STI_AS_-Am-V was significantly superior to La-V and STI_AS_-La-V (P < 0.001) with an AUC of 0.880, and a cutoff value of 0.05 μV providing 78.9% sensitivity and 92.0% specificity, while the AUC for Am-V and STI_AS_-La-V were both below 0.7 with values of 0.688 and 0.664 respectively, and there was no significant difference between them (P = 0.203) (Fig. 3).

**Table 3:** Univariate and multivariate analyses of standardized BAEPs for predicting postoperative hearing preservation.

Variables	Univariate analysis			Multivariate analysis		
	OR	(95% CI)	*P* value	OR	(95% CI)	*P* value
Raw BAEPs						
La-I (ms)	0.573	(0.120–2.736)	0.485	–	–	–
La-II (ms)	0.819	(0.218–3.074)	0.767	–	–	–
La-III (ms)	0.420	(0.117–1.503)	0.182	–	–	–
La-IV (ms)	0.647	(0.215–1.947)	0.439	–	–	–
La-V (ms)	0.353	(0.104–1.199)	0.095	–	–	–
La-I-III (ms)	0.583	(0.160–2.124)	0.414	–	–	–
La-III-V (ms)	0.798	(0.302–2.107)	0.649	–	–	–
La-I-V (ms)	0.601	(0.227–1.589)	0.305	–	–	–
Am-V (μV)	1703.860	(26.749–108,530.745)	**< 0.001**	–	–	–
Am-I (μV)	2.591	(0.122–54.847)	0.541	–	–	–
Standardized BAEPs						
STI_AS_-La-I	1.232	(0.223–6.814)	0.811	–	–	–
STI_AS_-La-II	2.338	(0.185–29.489)	0.512	–	–	–
STI_AS_-La-III	3.671	(0.144–93.713)	0.431	–	–	–
STI_AS_-La-IV	3.745	(0.058–240.066)	0.534	–	–	–
STI_AS_-La-V	0.002	(0.000–0.386)	**0.021**	< 0.001	(0.000–0.035)	**0.003**
STI_AS_-La-I-III	0.352	(0.097–1.277)	0.112	–	–	–
STI_AS_-La-III-V	0.289	(0.065–1.285)	0.103	–	–	–
STI_AS_-La-I-V	0.033	(0.001–1.129)	0.058	–	–	–
STI_AS_-Am-V	1.919	(1.340–2.749)	**< 0.001**	2.085	(1.426–3.050)	**< 0.001**

Significant values are in bold.

### Intraoperative BAEPs difference

**Figure 4:**
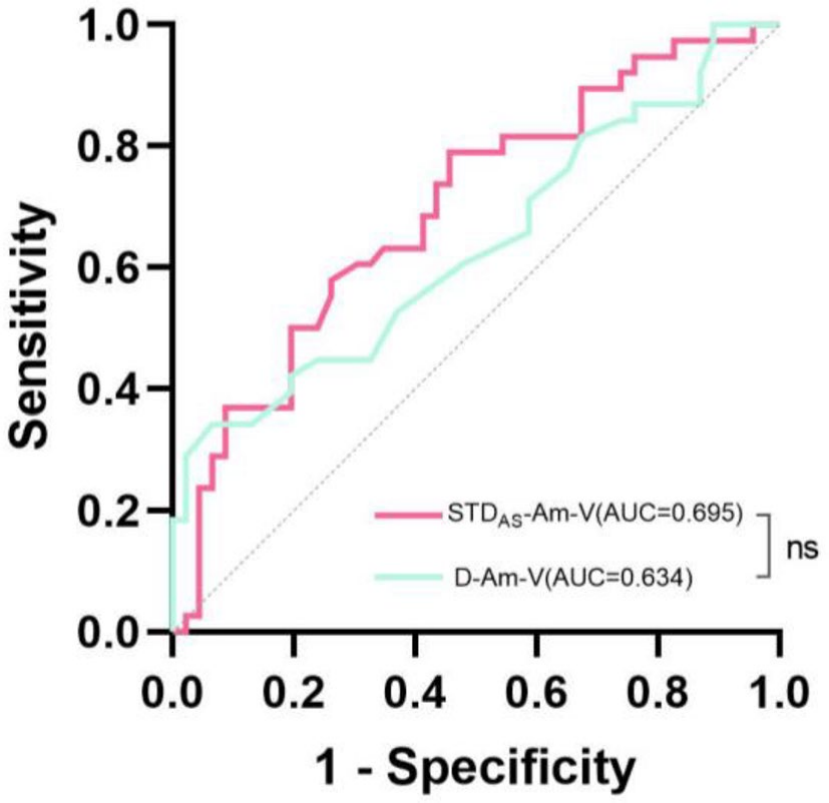
A receiver operating characteristic (ROC) curve of intraoperative BAEPs difference predictors of HP, ns No significant difference (P > 0.05).

Among the patients with preoperative HP, only 45.2% (38/84) still exhibited HP after surgery. Univariate logistic regression analysis was performed to assess the intraoperative BAEPs difference, revealing that D-Am-V (OR 242.018, P = 0.009) and STD_AS_-Am-V (OR 54.489, P = 0.02) were predictors of intraoperative HP (Table 4). ROC curve analysis demonstrated an AUC for D-Am-V was 0.634 with a cutoff value of 0.095 μV providing a sensitivity of 34.2% and specificity of 93.5%. The AUC for STD_AS_-Am-V was 0.695 with a cutoff value of 0 0.001 μV and displayed a sensitivity of 78 0.9% along with specificity of 54 0.3%. However, no significant difference in prediction accuracy between these two indicators was observed (P = 0.334) (Fig. 4).

**Table 4:** Univariate and multivariate analyses of intraoperative BAEPs difference for predicting hearing preservation.

Variables	Univariate analysis			Multivariate analysis		
	OR	(95% CI)	P value	OR	(95% CI)	P value
Intraoperative BAEPs difference						
D-La-I (ms)	0.273	(0.056–1.324)	0.107	–	–	–
D-La-II (ms)	0.837	(0.231–3.031)	0.787	–	–	–
D-La-III (ms)	0.601	(0.222–1.628)	0.317	–	–	–
D-La-IV (ms)	0.572	(0.236–1.384)	0.215	–	–	–
D-La-V (ms)	0.740	(0.258–2.122)	0.576	–	–	–
D-La-I-III (ms)	1.043	(0.344–3.166)	0.940	–	–	–
D-La-III-V (ms)	0.910	(0.395–2.097)	0.824	–	–	–
D-La-I-V (ms)	1.372	(0.883–2.132)	0.160	–	–	–
D-Am-V (μV)	242.018	(3.843–15,239.437)	**0.009**	–	–	–
D-Am-I (μV)	2.912	(0.116–72.816)	0.515	–	–	–
Standardized intraoperative BAEPs difference						
STD_AS_-La-I(ms)	0.379	(0.108–1.331)	0.130	–	–	–
STD_AS_-La-II(ms)	1.036	(0.428–2.507)	0.938	–	–	–
STD_AS_-La-III(ms)	0.903	(0.384–2.122)	0.815	–	–	–
STD_AS_-La-IV(ms)	0.875	(0.419–1.825)	0.722	–	–	–
STD_AS_-La-V(ms)	0.843	(0.342–2.078)	0.710	–	–	–
STD_AS_-La-I-III(ms)	1.551	(0.585–4.113)	0.378	–	–	–
STD_AS_-La-III-V(ms)	0.853	(0.492–1.477)	0.569	–	–	–
STD_AS_-La-I-V(ms)	1.471	(0.912–2.371)	0.113	–	–	–
STD_AS_-Am-V(μV)	54.489	(1.896–1566.114)	**0.020**	–	–	–
STD_AS_-Am-I(μV)	0.929	(0.502–1.719)	0.814	–	–	–

Significant values are in bold.

## Discussion

Recent technological developments, particularly the increased availability and accuracy of magnetic resonance imaging (MRI), have increased the detection rate of small VSs (less than 1.5 cm in diameter)^1^. When the tumor shows a slow/null pattern of growth, the wait-and-scan approach is appropriate, but irreversible hearing loss will still progress regardless of imaging evidence of tumor growth^20,21^. Gamma knife radiotherapy may also be considered for small VSs, which is a less invasive option than surgical resection. Although some authors report good short-term functional outcome and satisfactory functional preservation of the facial nerve in up to 95–100% of cases, tumors are not eradicated and disease control requires long-term evaluation^22,23^. Carlson et al. reported a hearing preservation rate of only 23% at 10 years after radiotherapy^24^. Surgical resection remains the only radical treatment. The protection of the cochlear nerve is still a difficulty in surgical resection for nearly 40% of patients suffer from hearing loss due to the injury of cochlear nerve during operation, however, surgical resection can achieve complete resection of the tumor and preserve the anatomical structure of the nerve^6,25^. The incidence of surgery-related non-nerve-related serious complications and facial nerve injury has been effectively controlled, and some studies even suggest that surgical treatment can provide a better chance of long-term hearing preservation than wait-and-scan approach^3,26^.

The dilemma of low HP rates following VSs surgery necessitates the implementation of reliable intraoperative cochlear nerve monitoring technology.

BAEPs, the most commonly employed intraoperative neuromonitoring technique for assessing cochlear nerve function during VSs surgery, records I–V waves corresponding to the auditory pathways in the brainstem. Cochlear nerve injury results in delayed latency and decreased amplitude of the V-wave. Most clinical neuroelectrophysiologists involved in BAEPs monitoring during CPA operations consider V-wave latency and amplitude as optimal electrophysiological indicators indicative of cochlear nerve injury caused by the operation^27^. However, previous studies have demonstrated that that these raw indicators lack reliability, a concern also reflected in our analysis findings^9^. Comparing pre- and post-resection raw BAEPs analysis results revealed that neither Am-V nor La-V can serve as consistent predictors of HP simultaneously, with both exhibiting an AUC value below 0.7. This is primarily blame to the low SNR of BAEPs, rendering them highly susceptible to interference.

Noritaka Aihara et al. attempted to mitigate parts of the interferences by utilizing healthy side BAEPs as a reference, resulting in improved prediction accuracy^28^. However, the authors solely obtained healthy side BAEPs within the laboratory setting, which failed to eliminate potential interferences from various internal and external environmental factors during long-term operation. This limitation significantly impacts the reliability of their conclusions; thus, it is recommended to acquire simultaneous healthy side BAEPs during operation.

In this study, we recorded binaural BAEPs continuously during the operation, and employed synchronous (interval between affected and healthy side ≤ 3 min) data from the healthy side as a reference for standardizing affected side BAEPs, thereby maximizing interference elimination.

As anticipated, standardized V-wave latency (STI_AS_-La-V) and amplitude (STI_AS_-Am-V) demonstrated consistent predictive ability for HP both pre- and post-resection, with an AUC exceeding 0.8 for STI_AS_-Am-V, indicating its high accuracy in predicting HP. Furthermore, we observed that the intraoperative difference in V-wave amplitude (D-Am-V) predicted postoperative HP in patients with preoperative HP (AUC 0.634) and remained predictive after standardization (STD_AS_-Am-V) (AUC 0.695).

The waveform of BAEP is usually composed of multiple positive and negative waves, each of which represents the activation of a different part of the auditory pathway. For example, wave I originates from the peripheral part of the cochlear nerve and reflects the action potential of the extracranial segment of the auditory nerve. Wave III is closely related to the electrical activity of the upper olive nucleus. The V-wave originated in inferior colliculus^17^. Usually, the tumor does not directly damage this anatomical structure, but rather causes an effect on the auditory conduction pathway, which is reflected from the pre-resection STIAS-La-III-V extension on the tumor side. When the tumor compresses the internal ear, it may also cause cochlear degeneration, as evidenced by changes in wave I recorded at the level of the first segment of the cochlear nerve, which explains the prolongation of pre-resection STIAS-La-I in our results^29^. V-wave latency and amplitude are widely recognized indicators of BAEPs during VSs surgery^19^. Our study highlights the importance of comparing intraoperative synchronous binaural V-wave amplitude to predict postoperative HP, particularly in patients with preoperative HP. While measuring BAEPs intraoperatively is a routine procedure, our findings represent the first report demonstrating that comparing simultaneous binaural V-wave amplitudes can serve as a reliable predictor of HP during VSs surgery.

There are certain limitations in this study. First, as a single-center study with limited number of cases, selection bias cannot be completely excluded, and larger sample size and multi-center data are still needed for verification. Secondly, this study is a retrospective study, and there may be unmeasurable confounding factors such as patient age, gender and tumor size that may be influencing factors of BAEPs, which need to be further explored in prospective studies. Finally, despite our efforts to standardize procedures and minimize interference, it is important to acknowledge the inherent limitations of BAEPs—in particular, their small amplitude (0.1–0.5 μV) makes repeated measurements (1000–2000 times) necessary on the one hand, resulting in long response delays (1–2 min), and makes BAEPs vulnerable to interference on the other hand. Future research should prioritize the exploration of novel cochlear nerve monitoring techniques that offer enhanced sensitivity and specificity. For instance, Daniele Starnoni et al. successfully monitored cochlear nerve function by directly stimulating the cochlear nerve to evoke postauricular muscle response, yielding promising HP results^30^. The challenge of low HP rates in VSs surgery will be overcome through advancements in neuromonitoring technology.

## Methods

### Patients

We conducted a retrospective review of all VSs patients who underwent surgical resection in our department from January 2018 to June 2022. The clinical, imaging, histopathological, operative reports, and outpatient records were obtained from the hospital information system and follow-up. In this study, we utilized Koos grades for preoperative classification of VSs^31^. The extent of surgical resection was determined based on magnetic resonance imaging (MRI) findings at 3 months postoperation^32,33^. Pure tone average (PTA) and word recognition scores (WRS) were assessed within 1–7 days before surgery and approximately 2 weeks after surgery. The results were evaluated according to the American Academy of Otolaryngology-Head and Neck Surgery (AAO-HNS) guidelines^34^. Primary outcomes were HP rates, defined as AAO-HNS classic A–C (PTA > 50 dB and WRS ≥ 50%). Patients were categorized into two subsets: those with HP and those without. All operations were performed via retrosigmoid craniotomy. Patients with bilateral VSs or hearing loss in the unaffected ear were excluded. The study was conducted in accordance with the Declaration of Helsinki, and the study protocol and a waiver of written informed consent were approved by the Clinical Research Center of Tongji Hospital (study approval no.: 202312573) and the Ethics Committee of Tongji Hospital (ethics approval no.:TJ-IRB20210523).

### Intraoperative BAEPs

During surgery, continuous monitoring of binaural BAEPs was conducted using a commercially available electrode system (Cadwell Medical, USA). A plug-in earphone was used to give sound, the stimulus sound was broadband clicking sound, and alternating wave stimulation was adopted, the stimulation frequency was 11.9 Hz, the stimulation intensity was 100 dB, the average signal frequency was 2000 times. Recording electrodes: Hypodermic needle electrodes, crown positive electrode (Cz), mastoid negative electrode on both sides (A1, A2). After anesthesia, positioning and fixation of the head frame, BAEP monitoring was performed until the end of surgery. BAEPs indicators encompassed I, II, III, IV, V, I–III, III–V, I–V waves latency, and I and V waves amplitude (measured from positive peak to next negative peak). Key surgical events such as dural incision or beginning/ending of tumor resection were simultaneously marked. Pre-resection BAEPs referred to recordings obtained from the start of surgery until tumor exposure while post-resection BAEPs represented recordings acquired between complete tumor resection and completion of surgery. Data pairing was performed based on affected and healthy sides with a criterion that the recording interval between affected and healthy side should not exceed 3 min. Paired BAEPs were standardized using the healthy side as a reference as follows:

$$ {\text{Standardized value of Affected Side }} = \frac{{\text{affected side value}}}{{\text{healthy side value}}} * {\text{ normal reference value}} $$

$$ {\text{Differencevalue in Affected Side }} = {\text{ affected side value}} - {\text{normal reference value}} $$

$$  \begin{gathered} {\text{Standardized difference value in Affected Side }} = {\text{ Standardized value of Affected Side}} \hfill \\ \quad - {\text{normal reference value }} = {\text{ normal reference value }}* \, \left( {\frac{{\text{affected side value}}}{{\text{healthy side value}}}{-1}} \right) \hfill \\ \end{gathered} $$

Considering that the normal reference is constant, we define the variable in above equation as the Standardized Index for Affected Side (STI_AS_), namely:

$$  {\text{STI}}_{{{\text{AS}}}} = {\text{Standardized Index of Affected Side }} = \frac{{\text{affected side value}}}{{\text{healthy side value}}}{-1} $$

Standardized intraoperative Difference value in Affected Side BAEPs (STD_AS_) are calculated according to following formula:

$$  {\text{D}}_{{{\text{AS}}}} = {\text{intraoperative Difference value in Affected Side }} = {\text{ affected side value}}_{{{}{\text{pos - resection}}{}}} {-}{\text{affected side value}}_{{{}{\text{pre - resection}}{}}} $$

$$ {\text{STD}}_{{{\text{AS}}}}  = {\text{Standardized}}{\mkern 1mu} {\text{intraoperative Differencevaluein Affected Side }} = {\text{D}}_{{{\text{AS}}}}  - {\mkern 1mu} ({\text{healthy side value}}_{{{\text{pos - resection}}}}  - {\text{ healthy side value}}_{{{\text{pre - resection}}}} ) $$

### Statistical analyses

The statistical analyses were conducted using SPSS statistical package software (version 26.0, IBM Inc., Chicago, US). Continuous variables were presented as mean ± standard deviation, and categorical variables were described as counts (percentage). One-way ANOVA was used for comparison between three or more groups, and paired sample T-test was used for comparison between two groups. Univariate binary logistic regression models were developed to identify predictive variables for HP. HP was considered the dependent variable, and the raw and standardized BAEPs indicators served as independent variables. Variables with a significance level of P ≤ 0.05 in the univariate analysis were included in the stepwise multivariate binary logistic regression model to determine independent predictors of HP. The results are presented with corresponding P values, odds ratios (ORs), and 95% confidence intervals (CIs). Receiver operating characteristic (ROC) curves were generated to evaluate the area under the curve (AUC) and determine cutoff values. ROC curve analysis was performed among different predictors for the same outcome measure. Statistical significance was defined as P < 0.05.

## Data Availability

Data are available on request and provided by corresponding author Kai Shu (kshu@tjh.tjmu.edu.cn).
